# Effect of the Microalga *Chlorella fusca* CHK0059 on Strawberry PGPR and Biological Control of Fusarium Wilt Disease in Non-Pesticide Hydroponic Strawberry Cultivation

**DOI:** 10.4014/jmb.2001.01015

**Published:** 2020-02-21

**Authors:** Min-Jeong Kim, Chang-Ki Shim, Byong-Gu Ko, Ju Kim

**Affiliations:** Organic Agricultural Division, National Institute of Agricultural Sciences, Wanju 55365, Korea

**Keywords:** *Chlorella fusca* CHK0059, *Fusarium oxysporum* f. sp. *fragariae*, Plant growth promoting rhizobacteria, strawberry

## Abstract

The purpose of this study was to identify strawberry wilt pathogens and evaluate the efficacy of *Chlorella fusca* CHK0059 for improving plant growth and suppressing Fusarium wilt. We identified 10 isolates of wilt pathogens of non-pesticide Seolhyang strawberry plant, including *Fusarium oxysporum* f. sp. *fragariae*, using morphological and molecular analysis. On the 15^th^ day after 0.4% CHK0059 treatment, the plant height of the untreated control strawberry plants was significantly greater than that of the CHK0059-treated strawberry plants. After 85 days, both treatments showed a similar tendency regarding the height of the strawberry plants. However, the thickness of strawberry leaves treated with the CHK0059 was found to be 1 mm thicker than that of the untreated control. The flowering percentage of the CHK0059 plants was also 40.2% higher on average than that of the untreated control. The chlorophyll content of strawberry leaves treated with the CHK0059 was also, on average, 6.63% higher than that of the untreated control. After 90 days of the CHK0059 treatment, the incidence of Fusarium wilt in the CHK0059-treated plants had reduced by 9.8% on average compared to the untreated control. The population density of *F. oxysporum* f. sp. *fragariae* was also reduced by approximately 86.8% in the CHK0059-treated plants by comparison to the untreated control at 70 days after treatment. The results indicate that the microalga *C. fusca* CHK0059 is an efficient biological agent for improving strawberry plant growth and suppressing Fusarium wilt disease in organic strawberries.

## Introduction

Strawberry (*Fragaria* × *ananassa* Duch) is an economically important crop; in Korea, annual strawberry output accounts for approximately 1.3 trillion won [30]. Many countries are cultivating strawberries on a large scale, with regard to which Korea ranks 6th in scale cultivation area worldwide and exports approximately 23 million tons of strawberries annually [27].

*Fusarium oxysporum* f. sp. *fragariae*, the causal agent of crown and root rot in strawberry plants, is one of a number of major limiting factors that lead to economic losses in strawberry cultivation fields and nursery facilities [52].

*F. oxysporum* f. sp. *fragariae* forms or produces three types of conidia, of which the microconidia are the smallest conidia. These are followed by the macroconidia, which are known to cause disease. The largest conidia are the chlamydospores, which are resistant to adverse environment conditions [39]. Among various genetic markers, the ITS and β-tubulin genes have been evaluated as suitable for discriminating between species of *Fusarium* [41].

Fusarium wilt pathogens penetrate through and browse strawberry roots, and then grow in the ducts of the crown and block the conduits to block the movement of nutrients, forming new and yellow leaves and eventually causing the death of the plants [32, 38]. Strawberry wilt has occurred in the ‘Dochiodome’ and ‘Redpearl’ strawberry varieties bred in Japan [33, 43]. In addition, it has been reported that the disease has damaged many ‘Meyhyang’ and ‘Huemhyang’ strawberry varieties bred in Korea [36]. Recently, ‘Akihime’, which has been recognized as a resistant cultivar, has also widespread wilt disease. Among the strawberry varieties cultivated domestically, 83.4% of the ‘Seolhyang’ strawberry variety plants were affected by the disease [27]. The prevalence of Fusarium wilt disease is increasing along with increasing soil cultivation, especially hydroponic cultivation, in Korea. The Fusarium wilt disease is related to pH and electrical conductivity (EC) in hydroponic culture of strawberry plants. The incidence of Fusarium wilt disease has been shown to be highest at the pH 5 level and increased as the EC increased in strawberry field trials [37].

Since Strawberry Fusarium wilt disease is difficult to control once it has occurred, lowering the disease incidence is the most important control management strategy [46, 48]. Strawberry wilt may be due to soil-borne infectious diseases, which can survive for long periods of time under natural conditions [26, 44].

Standard integrated pest management practices including crop rotation with non-hosts, the planting of pathogen-free transplants and the sanitation of equipment remain important measures that can reduce the risk of damage from Fusarium wilt [32, 36]. However, to control strawberry Fusarium wilt, the application of disease-resistant cultivars against Fusarium wilt is the most promising management for sustainable agricultural practices [34].

Red and brown algae are mainly used as healthy food, source for humans due to their high concentration of polysaccharides and natural richness in minerals, polyunsaturated fatty acids and vitamins [13, 51]. Chlorophyceae members including *Chlorella* have also been explored as biofertilizers, as they are rich in carbohydrates, proteins, lipids, and growth hormones. A wide variety of macro algal extracts have been explored as bio-stimulants in conventional agriculture. Among these microalgae, *Chlorella vulgaris* [10, 14, 45], *Chlorella pyrenoidosa* [2], *Acutoseamua dimorphus* [16], *Spirulina platensis* [10] are considered to be good candidates, as all are able to increase the growth and yield of plant. The field applications of microalgae have included its use as a biofertilizer for various crops, including mango [1], grape [5], tomato [16, 45], potato [29], Chinese chives [22], cucumber and eggplant [2], lettuce [2, 14], okra [3], spinach [22], wheat [17] and corn [10].

Antifungal properties have also been reported to exist in various algal extracts, such as *Anabaena* spp. [15, 31], *Chlorella* sp. [22, 47], *Scenedesmus* sp. [4], *Scytonema* spp. [8, 19] and *Nostoc* spp. [6]. In addition, several researchers have suggested that algal extracts significantly inhibit the plant fungal pathogens, *Botrytis cinerea* [12], *Colletotrichum orbiculare* [23] and *Magnaporthe grisea* [7].

Recently, freshwater algae, also known as *Chlorella fusca* CHK0059, which was already known to have positive impacts on the growth and qualities of organic Chinese chives and spinach [22] was shown to induce systemic acquired resistance in cucumber plants against anthracnose caused by *C. orbiculare* [23].

The aim of the present study was to identify strawberry wilt pathogens and investigate the effects of *C. fusca* CHK0059 on improving strawberry plant growth and suppression of Fusarium wilt caused by *F. oxysporum* f. sp. *fragariae*. The study was conducted at a hydroponic strawberry cultivation farm certified as non-pesticide.

## Materials and Methods

### Planting and Cultivation

The strawberry growth promotion effect and the wilt disease suppression effect of chlorella treatment in strawberry hydroponics were investigated from October 2017 to May 2018 at an organic strawberry cultivation farm (GPS location: 36.221637, 127.215531) in Nonsan. The artificial soil used for the test was a strawberry ground soil (coco peat: peat moss: pearlite = 65: 17: 10, Seoul Bio). The cultivation medium was adjusted to EC and pH according to the cultivation method typically used by farmers using raw fertilizer (NPK: 30-10-10, 1000 times) and EC (0.63), respectively. We used 30 strawberry plants per one replications of each experiment in this study.

### Isolation and Culture of Fusarium Wilt Fungus

Cultures of *F. oxysporum* f. sp. *fragariae* were obtained from infected strawberry plants collected from a hydroponic strawberry cultivation bed in Nonsan and were grown in Komada’s selective medium [25] at 25°C for 7 days. Fusarium colonies were identified on the basis of their cultural characteristics and transferred to potato dextrose agar (PDA; potato infusion 200 g, glucose 20 g, agar 15 g). Each *F. oxysporum* f. sp. *fragariae* isolate was grown in potato dextrose broth at 25°C for 4 days. Fungal mycelia was harvested from the liquid medium, rinsed with sterile distilled water, blotted to remove excess liquid, and frozen in a -70°C deep freezer for 3 h. Following lyophilization, the genomic DNA was extracted from 10 mg of lyophilized mycelia for molecular identification of the strawberry Fusarium isolate NSS02. Species identification was confirmed by using either microscopic morphological characteristics and a molecular method.

### Morphological and Molecular Characterizations of Fusarium Wilt Isolate

For morphological identification, single spore isolates were grown for 10 days on PDA medium. Microscopic features of micro and macro conidia and chlamydospores were also determined based on Nelson *et al*. (1983) and Summeral *et al*. (2003) [40, 50]. Molecular identification of Fusarium wilt isolate was carried out based on conserved ribosomal internal transcribed spacer (ITS) regions [41, 52] and β-tubulin encoding genes [18, 41]. We amplified the ITS regions between the small nuclear 18S rDNA and large nuclear 28S rDNA, including the region of 5.8S rDNA, using primer pair ITS1 and ITS4, as shown in Table 1. A fragment of the β-tubulin encoding gene (btu2) was amplified with primer pair T10 and T224, as shown in Table 1. Amplification was performed using a Thermal Cycler (Applied Biosystems TC1) with a 25 μl reaction volume. Each reaction mixture contained a 2 μl 10× buffer with magnesium chloride (MgCl_2_), 0.5 μl of 10 mM dNTPs, 2 μl of 10 mMUP-PCR primer, 2 units of Taq DNA polymerase (TaKaRa Hot-Taq, Japan) and 2 μl of DNA template (50–100 ng), and the volume was increased to 25 μl with double-distilled sterile water. PCR was performed for the ITS region using the following steps: (i) 94°C for 5 min; (ii) 30 cycles of 92°C for 60 sec, 58°C for 60 sec, and 72°C for 90 sec; and (iii) a final extension step of 72°C for 5 min. PCRs were performed for the β-tubulin encoding gene (btu2) using the following steps: (i) 94°C for 5 min; (i) 30 cycles of 92°C for 60 sec, 56°C for 60 sec, and 72°C for 90 sec; and (iii) a final extension step of 72°C for 15 min. The PCR products were run on 1.4% low melting agarose gels, stained with ethidium bromide (EtBr) and viewed under a UV transilluminator. For ITS and β-tubulin DNA fragment sequencing, template DNA (20 μl) was directly prepared from PCR products by purification using a column based purification kit (Qiagen, Germany). Sequencing was then performed using Chunlab Co. (Korea). Sequencing similarities were compared to sequences previously deposited in the National Center for Biotechnology database (NCBI, https://blast.ncbi.nlm.hih.gov/Blast.cgi). Molecular phylogenetic tree based on the amino acid sequence of the ITS and β-tubulin gene of isolate NSS02 was obtained automatically by applying Neighbor-Join algorithms to a matrix of pairwise in MEGA version 7 [30]. The matrix of pairwise was inferred by using the maximum composite likelihood (MCL) method, with a 1000-replicate boot strap test, using MEGA 7. The phylogenetic tree was drawn to scale, with branch lengths measured by the number of substitutions per site.

### Culture and Treatment of Chlorella

The Chlorella strain used in this study was *Chlorella fusca* CHK0059 isolated from an organic rice paddy field before being cultured to purify in our laboratory [21]. The culture of chlorella was prepared by adding 5 ml of chlorella exclusive culture medium (F & B Nature Co. Ltd., Korea) modified with Bold’s basal media and BG11 media [21] to 8 L of commercial mineral water, and then inoculating uncontaminated *C. fusca* using “the chlorella farmer’s self-light incubator” at the farmer’s greenhouse in Nonsan. After inoculation, artificial light was irradiated over the chlorella at more than 2,000 lux, approximately equal to sunlight at 28~30°C, while air was blown over 5 L/min using a home bubble generator. The chlorella culture solution was left for 5 to 7 days; it was then examined using a light microscope (Leica, DM5500B, Japan) with a hemocytometer (Marienfeld, Germany). The chlorella culture solution which the concentration of *C. fusca* CHK0059 was 1.5 × 10^7^ cells/ml or more was used in this experiment. The chlorella treatment was diluted with water to a concentration of 0.4%. The control was sprayed with water, followed by leaf application and soil irrigation, every two weeks using a high-pressure sprayer.

### Estimation of Plant Growth and Fusarium Wilt

From the 15th day after the 0.4% chlorella CHK0059 treatment, the plant height, chlorophyll content, leaf thickness (Micrometers, Multitoyo, Japan) and flowering of the strawberry plants were investigated. Chlorophyll content (SPAD) was measured from sample leaves of plants grown under light at each treatment time using a CM-100 Chlorophyll meter (Spectrum Technologies Inc., USA). The average SPAD value of three readings obtained from each leaves was used.

The incidence of spontaneous strawberry Fusarium wilt disease was examined at each treatment on 13 day intervals from September 28 to November 6. A comparison between two treatments was used to calculate the mean and standard error of each treatment.

### *F. oxysporum* f. sp. *fragariae* Population Density in the Tested Nursery Medium

Population densities of *F. oxysporum* f. sp. *fragariae* were determined using a dilution plate technique after each treatment. Approximately 10 g of the tested rhizosphere strawberry soil from each test plot was placed in a 250 ml glass flask containing 90 ml sterile distilled water. After being shaken for 60 min at 200 rpm by a rotary shaker at room temperature, the suspension was serially diluted in 9 ml of sterile distilled water. Then, 1 ml of the appropriate dilution of each of three replicates was spread on Komada’s selective medium [25] and stored at 25°C in the dark for seven days. The number of colonies was then counted and recorded as colony forming units (CFU) per gram of dry soil sample.

### Statistical Analysis

The gathering of experimental data was repeated a minimum of three times. Student’s *t*-test was conducted to compare the isolates of Fusarium wilt pathogens using Microfost Excel software. The plant growth characteristics, plant height, leaf thickness, SPAD, and flower number, and the disease incidence and population density of Fusarium wilt pathogens were subjected to statistical analysis using SAS program for windows version (ver. 9.2_PC32, SAS Institute Inc., USA). Significance testing between treatments was analyzed using the least significant difference (LSD) at a 5% level.

## Results

### Isolation and Identification of Strawberry Fusarium Wilt Pathogens

**Fig. 1:**
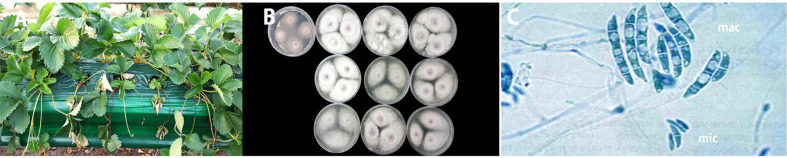
Symptoms of the ‘Seolhyang’ strawberry plant (A), mycelia growth of wilt pathogens on PDA (B), and microscopic observations (C) of macroconidia (mac) and macroconidia (mic) of wilt pathogens caused by *Fusarium oxysporum* f. sp. *fragariae*.

Diseased strawberry samples with Fusarium wilt were collected from the hydroponic high bed strawberry cultivation farm certified with non-pesticide level in Nonsan in 2017. Both wilt symptoms and whole strawberry plant collapse, which took place from September 7 to October 31, were observed to a high degree in the untreated control plants. The severely diseased crown of the ‘Seolhyang’ strawberry exhibited a brown to orange-brown discoloration (Fig. 1A). Ten of Fusarium-like isolates with light purple mycelia and orange reverse colony colors were isolated from the crowns of dead or dying untreated control plants (Fig. 1B). Macroconidia were 2 to 5 septate, straight to slightly curved, gently tapered and curved at the apical end (14.0 to 73.7 × 3.8 to 9.9 μm). Microconidia were oval ellipsoid, 0-septate and formed abundantly on short monophialides (8.6 to 22.2 × 3.8 to 7.7 μm) (Table 2; Fig. 1C). Chlamydospores were oval ellipsoid, 0-septate and formed abundantly on short monophialides (7.8 to 33.1 × 7.7 to 34.5 μm; Table 2).

**Fig. 2:**
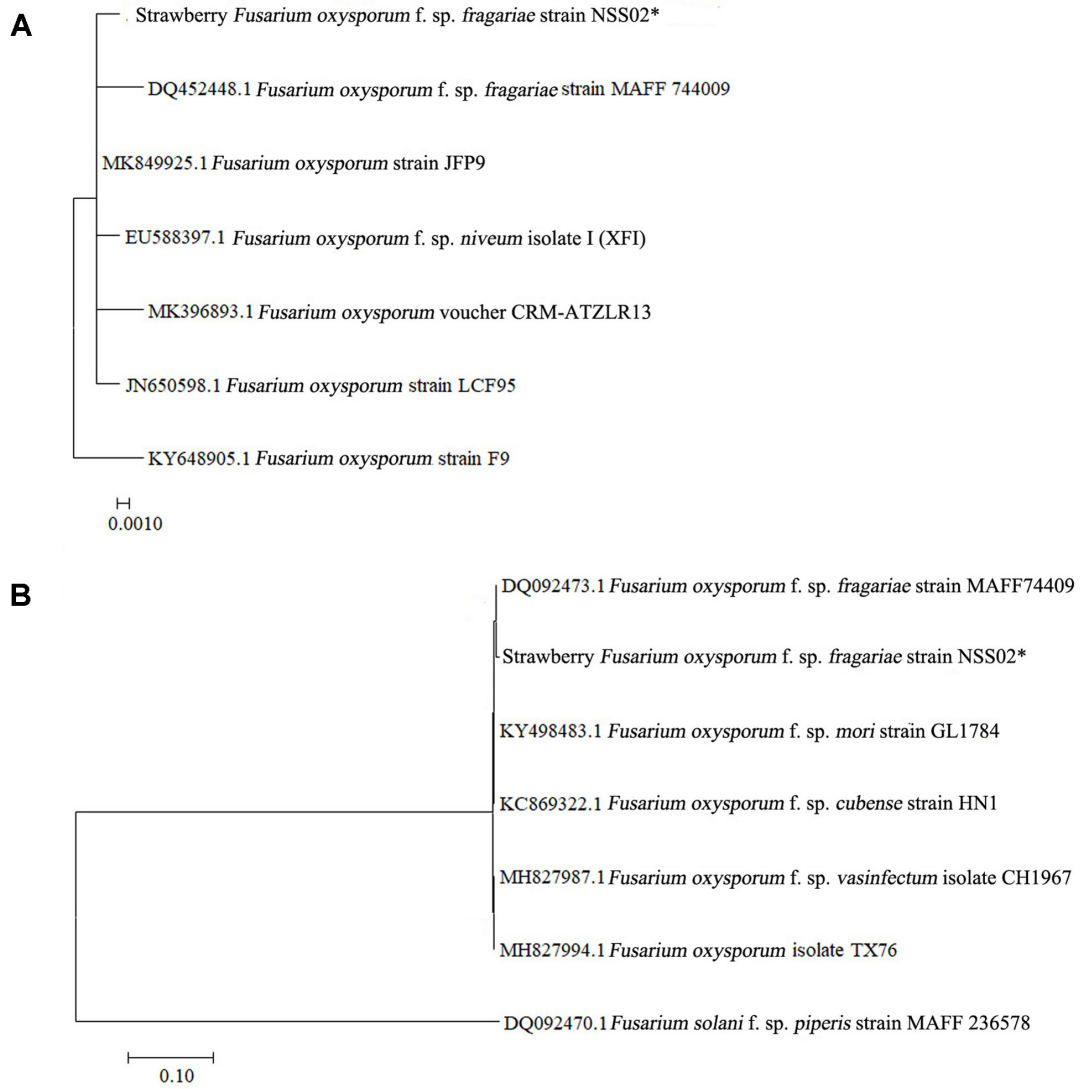
Molecular phylogenetic tree off Fusarium wilt isolate NSS02 in the Seolhyang strawberry constructed using the partial nucleotide sequences of the 18S rDNA-5.8S rDNA (A) and the -tubulin gene (B). Phylogenetic tree analysis based on the maximum composite likelihood (MCL) substitutions per site. Each node indicates bootstrap percentage based on 1,000 replications of the MCL. The scale bars means the average distance between clusters.

In order to support the identification results based on the morphological characteristics of the Fusarium wilt pathogens, the genetic sequences of the NSS02 strains were obtained for analysis of their molecular relationships in this study. PCR experiments were conducted on the nuclear rDNA region; 18S rDNA-5.8S rDNA reproducibly amplified a fragment of approximately 545 bp, using the primer pair ITS1 and ITS4. Discordant genetic trees were obtained from contiguous nuclear 18S rDNA-5.8S rDNA loci (Fig. 2A). The 18S rDNA-5.8S rDNA sequence analysis of the NSS02 isolate isolated from the strawberry showed 100% homology with the *F. oxysporum* f. sp. *fragariae* MAFF 744009 strain, a strain previously registered in GenBank (http://ncbi.nlm.nih.gov). In addition, phylogeny of the NSS02 isolate belonged to the *F. oxysporum* species complex, which includes *F. oxysporum* f. sp. *fragariae* and *Fusarium oxysporum* f. sp. *niveum* (Fig. 2A). PCR experiments conducted on the β-tubulin encoding gene *BTU2*, reproducibly amplified a fragment of approximately 616 bp, using the primer pair T10 and T224. Discordant genetic trees were obtained from contiguous nuclear β-tubulin loci (Fig. 2B). The β-tubulin gene sequence analysis of the NSS02 isolate isolated from the strawberry showed 100% homology with the *Fusarium oxysporum* f. sp. *fragariae* MAFF 744009 strain, a strain previously registered in GenBank. In addition, phylogeny of the NSS02 isolate belonged to the *Fusarium oxysporum* species complex including *Fusarium oxysporum* f. sp. *fragariae*, *Fusarium oxysporum* f. sp. *mori*, *Fusarium oxysporum* f. *cubense* and *Fusarium oxysporum* f. sp. *vasinfectum* (Fig. 2B).

### Enhancing Strawberry Plant Growth

**Fig. 3:**
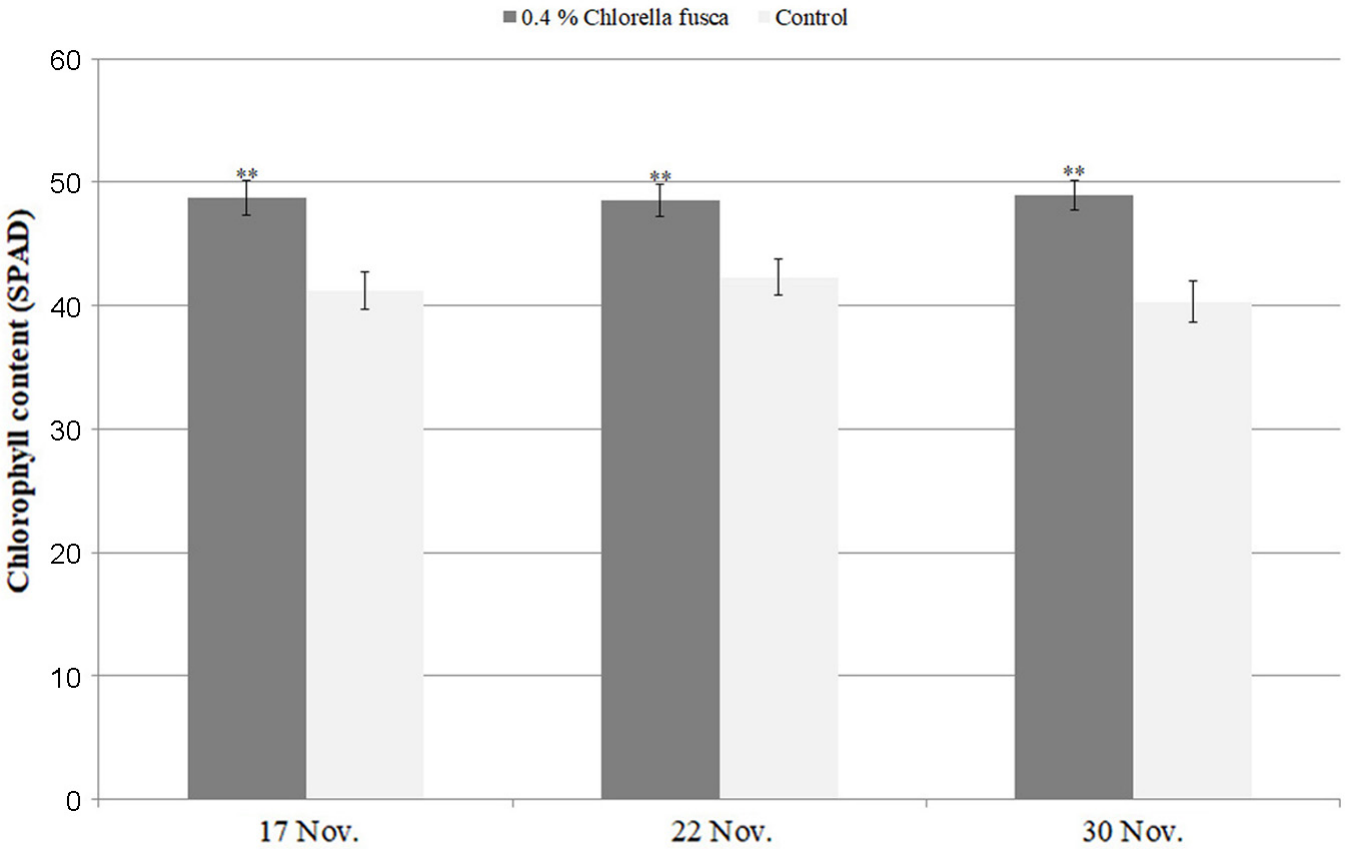
Comparison of chlorophyll content (SPAD) in Seolhyang strawberry leaves treated with 0.4% *Chlorella fusca* and untreated controls. **Correlation significance is at *p* < 0.001.

**Fig. 4:**
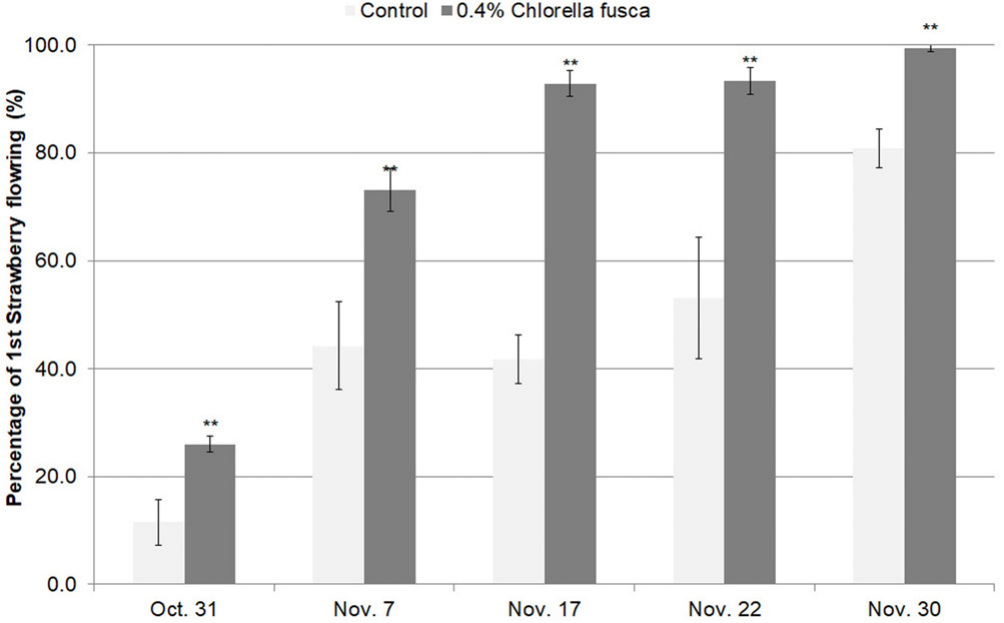
Improving flower number of Seolhyang strawberry plants treated with 0.4% *Chlorella fusca* at first flowing period on a hydroponic high bed in Nonsan. *Correlation significance is at *p* < 0.05, **Correlation significance is at *p* < 0.001.

To investigate the effect of the chlorella on plant growth, we calculated the height and leaf thickness of the strawberry plants at 7 day intervals from October 31 to November 30. The height and leaf thickness of the plants treated with 0.4% chlorella CHK0059 treatment significantly increased compared with those of untreated control. In particular, the leaf thickness of the chlorella treatment plants increased 16.6% by October 31, while that of untreated control decreased 6.1% over the same period (Table 3). The chlorophyll content (SPAD) of the ‘Seolhyang’ strawberry leaves treated with 0.4% chlorella CHK0059 treatment was significantly on average 18%higher than that of untreated control from November 17 to November 30 (Fig. 3). The effect of chlorella treatment on strawberry flowering number was found to be 14.4~51% higher than that of untreated strawberry plants from October 31 to November 30 (Fig. 4).

### Suppression of Fusarium Wilt Disease in Strawberry Plants

After 30 days of 0.4% chlorella CHK0059 treatment, the effect of chlorella on strawberry wilt disease was investigated. The strawberry wilt disease had occurred at a 0.2% disease incidence rate in the both treatment on September 28. The symptoms of diseased strawberry plants showed both wilt and whole plant collapse. On November 6, there was a 14.2% disease incidence, with symptoms including the death or dying strawberry plants (Table 4 and Fig. 3). In the 0.4% chlorella CHK0059 treatment plants, strawberry wilt disease had a occurred at a 0.2% disease incidence rate on October 11, one week later than the same incidence rate for the untreated control. After 69 days of 0.4% chlorella CHK0059 treatment, there was 4.2% disease incidence in the plants, 10.0% lower than in the untreated controls on November 6. Overall, 0.4% chlorella CHK0059 treatment was found to inhibit Seolhyang strawberry wilt disease by 70.4% compared with the untreated control (Table 4).

### Decreasing of the Population Density of *Fusarium oxysporum* f. sp. *fragariae*

**Fig. 5:**
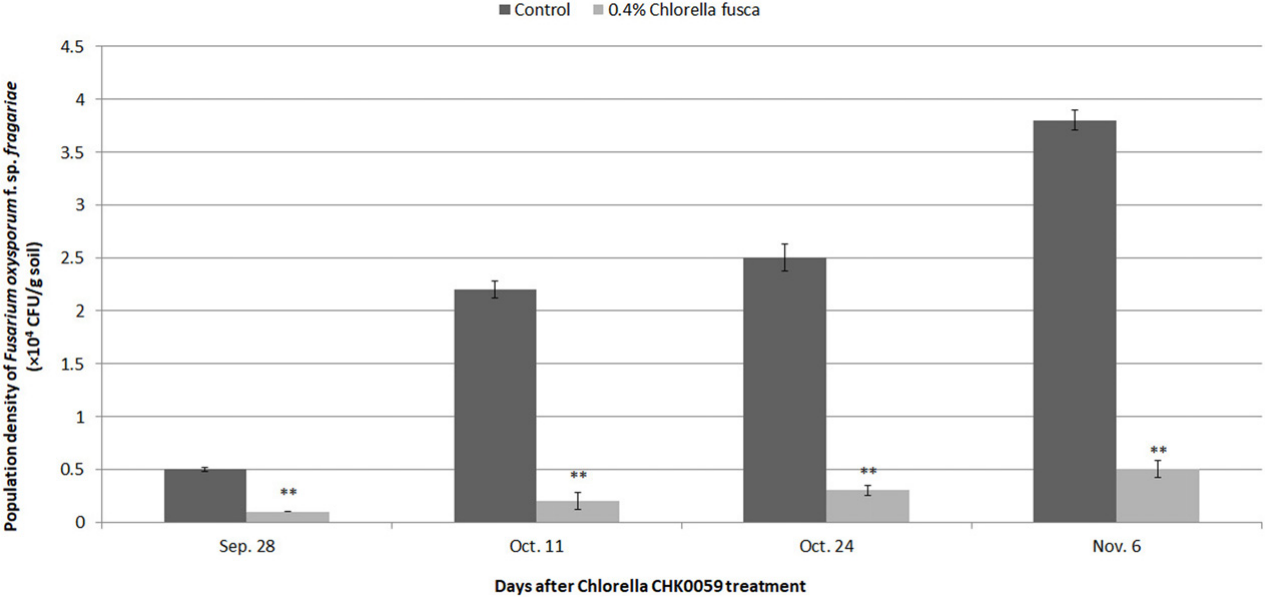
Effect of 0.4% *Chlorella fusca* CHK0059 on the soil population density of *Fusarium oxysporum* f. sp. *fragariae* in the hydroponic cultivation bed in Nonsan. DAT : days after treatment. Data are expressed as means ± SE (*n* = 30). **Correlation significance is at *p* < 0.001.

Treatments of an *F. oxysporum* f. sp. *fragariae* -infested artificial medium with 0.4% *C. fusca* CHK0059 resulted in significant reductions in the population densities (Fig. 5). Compared with the untreated control, an approximately 86.8% reductions was observed in the 0.4% chlorella CHK0059 treatment plants at 69 days after treatment (Fig. 5).

## Discussion

In our results, the symptoms and morphological characteristics of wilt disease in ‘Seolhyang’ strawberry plants were similar to the descriptions of *F. oxysporum* f. sp. *fragariae* given by Kim *et al*. (1982) and Cho and Moon (1984) [9, 20]. Then morphological identification was confirmed by amplification and sequencing of a partial fragment of the ITS region and the β-tubulin gene (*BTU2*) in Fig. 2.

We identified *Fusarium oxysporum* f. sp. *fragariae* based on the overall results from the morphological characterization of strawberry Fusarium wilt pathogen NSS02 isolated for one year in a non-pesticide strawberry cultivation hydroponic bed. The tested field was one in which hydroponic cultivation of strawberry plants began using new artificial medium and strawberry seedlings, but in which severe wilting disease had occurred. It can be estimated that the cause of the wilting disease was pathogens from seedlings infested with *F. oxysporum* f. sp. *fragariae*.

According to our results, the 0.4% *C. fusca* CHK0059 cultures used at two-week intervals, to treat the strawberry plants significantly enhanced and promoted plant growth promotion, including strawberry plant height and leaf thickness (Table 3), chlorophyll content (SPAD) (Fig. 3) and flowering number (Fig. 4).

*Chlorella vulgaris* has been shown to positively increase fresh and dry weight of lettuce seedlings [1, 14]. The irrigated application of *Chlorella pyrenoidosa* also increased the number of leaves and leaf surface area in soybean seedlings [11]. This is likely due to the fact that blue green alga produces plant growth promoting regulators that are similar to the plant hormones, gibberellin and auxin [54].

However, Zaccaro *et al*. (2001) reported that the foliar application of biochemical organic substances, which supply macro and micronutrients, has increased in popularity [54]. In the previous our study, the fresh weight and yield of the spinach treated with the chlorella CHK0059 was higher than that of the untreated. Also, the mineral content of K, Ca, Mg, P, Fe, and Mn in the chlorella CHK0059 was recorded higher than untreated control [22].

In addition, results obtained for algae indicate that the alga affects cell metabolisms, mainly through the physiological action of major and minor nutrients, amino acids, and vitamins. As well, it has been suggested that its growth regulators affect the cellular metabolism of treated plants, leading to enhanced growth and crop yield [1, 42, 49].

The dry biomass of microalgae, *Acutodesmus dimorphus*, is also able to induce the plant growth promotion and flowing number in Roma tomatoes [16]. Overall, the growth enhancement in the present study is similar to that revealed in these earlier reports.

*C. vulgaris* used as a biofertilizer on tomatoes has been shown to have a positive effect on plant growth, yield and other fruit qualities, such as dry weight and soluble solid content [4]. An extract from microalgae applied to soil or foliage has also been shown to increase ash, protein and carbohydrate contents of potatoes [29].

In recent years, there have been many reports of compounds derived from macroalgae that have a broad range of biological uses, including as antibacterial, antiviral, antioxidant, and antifungal against plant pathogens, *Magnaporthe grisea* [7], *Botrytis cinerea* [12, 22] and *Colletotrichum orbiculare* [23]. El-ghanam *et al*. (2015) reported that the bio agents, *Chlorella vulgaris* and *Spirulina platensis* can decrease liner growth and spore production of *Botrytis cinerea* in an open strawberry field. Their combination treatment caused a 0 disease severity (DS) % after a second spray when stored at 5°C [12]. Abd El Hafiz *et al*. (2015) reported that the control seedlings of cucumber were infected while infections were not induced in cucumber seedlings treated with *C. vulgaris* and *C. pyrenoidosa* [2]. Beena and Krishnika (2011) further reported that a freshwater microalga, *Scenedesmus* sp. is able to suppress three bacterial strains [4].

In conclusion, our results indicate that treatment using the microalgae strain, *Chlorella fusca* CHK0059 increases organic strawberry plant growth. It is suggested that, the *C. fusca* CHK0059 promoted the strawberry plants growth observed in this study. *Chlorella fusca* CHK0059 also increased the efficiency of controlling the strawberry Fusarium wilt disease through lowering the population density of *F. oxysporum* f. sp. *fragariae*. This remarkable reduction in disinfecting efficacy is probably due to competing reactions of *C. fusca* CHK0059 with organic substances in the artificial medium.

## Figures and Tables

**Table 1:** Target genes and PCR primer sets for the molecular identification of *Fusarium oxysporum* f. sp. *fragariae* isolated from Seolhyang strawberry plant in hydroponic cultivation bed.

Target genes ^y^	Primer	Primer DNA sequence (5‘ → 3‘)	References
ITS	ITS1	TCCGTAGGTGAACCTGCGG	[55]
	ITS4	TCCTCCGCTTATTGATATGC	
β-tubulin	T10	ACGATAGGTTCACCTCCAGAC	[43]
	T224	GAGGGAACGACGGAGAAGGTGG	[21]

^y^target genes : ITS , The small nuclear 18S rDNA and large nuclear 28S rDNA, including the region of 5.8S rDNA; -tubulin, The fragment of the -tubulin encoding gene (*btu2*).

**Table 2:** Characteristics of *Fusarium oxysporum* f.sp. *fragariae* NSS02 isolate obtained from Seolhyang strawberry plants caused by PDA medium.

Spore type	No. of Septum	Length of spore (µm)			Width of spore (µm)		
	
		Min	Max	Mean^a^	Min	Max	Mean
Microconidium	0-sept.	8.6	22.2	13.2±4.5	3.8	7.7	5.0±0.6	
Macroconidium	2-sept.	14.0	34.2	20.7±7.5	3.8	9.8	6.4±2.4	
	3-sept.	17.6	42.1	29.1±7.9	3.8	9.9	7.1±2.4	
	4-sept.	25.3	62.5	41.7±16.0	3.8	9.8	5.9±1.8	
	5-sept.	45.2	73.7	62.8±13.2	5.9	7.9	7.2±0.8	
Chlamydospore	-	7.8	33.1	15.6±7.2	7.7	34.5	15.2±7.9	

a : Data are expressed as means ± SE (*n* = 20).

**Table 3:** Comparison of height and leaf thickness of Seolhyang strawberry plants after being treated with 0.4% *Chlorella fusca* CHK0059 in hydroponic high bed cultivation in Nonsan.

Date	Treatment	Height (cm)	Leaf thickness (mm)
Oct. 31	0.4% *Chlorella fusca*	29.1±2.4^**^	3.6±0.01^**^
	Control	27.4±1.9	3.3±0.02
Nov. 7	0.4% *Chlorella fusca*	29.7±2.4^*^	3.6±0.05^**^
	Control	28.4±2.1	2.3±0.03
Nov. 17	0.4% *Chlorella fusca*	32.2±2.7^**^	4.1±0.04^**^
	Control	29.0±3.2	3.5±0.03
Nov. 22	0.4% *Chlorella fusca*	30.8±2.6^*^	4.2±0.05^**^
	Control	30.3±2.2	3.2±0.03
Nov. 30	0.4% *Chlorella fusca*	32.4±2.3^**^	4.2±0.04^**^
	Control	30.7±1.7	3.1±0.02

Data are expressed as means ± SE (*n* = 30).

*Correlation significance is at *p* < 0.05, **Correlation significance in at *p* < 0.001.

**Table 4:** Control effect of 0.4% *Chlorella fusca* CHK0059 on Seolhyang strawberry Fusarium wilt caused by *Fusarium oxysporum* f.sp. *fragariae* in hydroponic high bed cultivation in Nonsan.

Treatment	Disease incidence of Fusarium wilt (%)			

	Sep. 28	Oct. 11	Oct. 24	Nov. 6
Untreated control	0.2±0.1	5.3±0.8	9.1±0.8	14.2±0.6
0.4% *Chlorella fusca*	0.2±0.0	0.2±0.1 ^**^	2±0.2 ^**^	4.2±0.5 ^**^

Data are expressed as means ± SE (*n* = 30).

^**^Correlation significance is at *p* < 0.001.
